# Behavior of wild tigers in captivity at the Central Zoo, Nepal

**DOI:** 10.1371/journal.pone.0353056

**Published:** 2026-07-16

**Authors:** Nishan Pokhrel, Narayan Prasad Koju, Sandeepa Gautam, Randall C. Kyes

**Affiliations:** 1 GoldenGate International College, Tribhuvan University, Kathmandu, Nepal; 2 Center for Postgraduate Studies, Nepal Engineering College, Pokhara University, Nepal; 3 Department of Psychology, University of Washington, Seattle, Washington, United States of America; 4 Washington National Biomedical Research Center, University of Washington, Seattle, Washington, United States of America; 5 One Earth Institute, Seattle, Washington, United States of America; University Centre Sparsholt, UNITED KINGDOM OF GREAT BRITAIN AND NORTHERN IRELAND

## Abstract

The majority of tigers (*Panthera tigris*) maintained at the Central Zoo in Jawalakhel, Nepal represents rescues resulting from human-wildlife conflict situations. Their behavior in captivity can be influenced by a variety of factors including environmental and social variables such as visitor presence, sex of the tiger, and duration of captivity. In this study, we investigated how visitor presence, sex, and captivity duration influence the behavior pattern of captive tigers. Behavioral observations were conducted on four adult tigers (two males and two females) across summer and monsoon seasons using instantaneous scan sampling guided by a detailed ethogram and supported by CCTV recordings. Data were collected during both peak visiting hours and off-peak periods. Overall, the tigers exhibited high levels of inactivity, predominantly sleeping (43.7%) and resting (34.2%). Visitor presence significantly affected activity patterns of both male and female tigers (p < 0.001), marked by increased pacing and drinking behavior during peak hours. The off peak hours were dominated by resting and sleeping. Females tigers displayed more pacing and exploratory behaviors, while males were more sedentary. A recently rescued female (n = 1) exhibited higher level of play and exploration, whereas long-term captives showed elevated pacing and more repetitive and predictable routines (p < 0.001). Visitor presence, sex, and duration in captivity interactively shape the behavior expression and welfare indicators in these captive tigers. These findings highlight the importance of effective visitor management and environmental enrichment and play an important role in establishing welfare strategies to mitigate stress in captive tigers.

## 1. Introduction

Wild tigers (*Panthera tigris*) occasionally end up in controlled environments like zoos, rescue centers or sanctuaries. These animals may enter captivity for various reasons: rescued as a result of human-tiger conflict, confiscation from illegal trade, translocation due to habitat loss or for conservation and breeding purposes [1]. Most of the captive tigers maintained at the Central Zoo in Jawalakhel, Nepal was rescued from different protected areas and community forests as a result of human-wildlife conflict. These individuals, identified as “man-eaters” and implicated in human injury or fatality, were removed from the wild to ensure public safety [2].

When a tiger becomes man-eater, management authorities face limited options. Captivity is often viewed as a compromise between ensuring human safety and preserving the animal’s life [3]. However, the transition from a vast, complex natural habitat to a restricted zoo enclosure can profoundly alter an individual’s behavioral repertoire. In the wild, tigers engage in a diverse variety of natural behaviors like hunting, swimming, resting and regular territory patrolling. Following relocation to captivity, particularly after stressful conflict situations, tigers frequently display modified or abnormal behavioral patterns, including stereotypic pacing, altered feeding habits, and heightened responsiveness to human presence [4].

Such behavioral changes are closely linked to stress and compromised welfare. In large carnivores, stress often arises from the inability to engage in species-specific behaviors, limited spatial freedom, reduced hunting activities, and close proximity to humans [5]. For man-eating tigers, captivity may pose an additional psychological strain as a result of previous conflict experiences and potential trauma. Prolonged exposure to zoo visitors, noise and visual stimuli can act as a chronic stressor, influencing tiger behaviors during visiting hours, including resting, feeding, vocalizations, and aggression. In contrast, during off hours, these tigers display more relaxed and natural behaviors, such as resting or sleeping, suggesting that human presence can significantly influence their behavioral welfare [6].

Although the welfare of zoo-housed animals has become a growing concern, limited research has examined the behavioral impact of human presence on captive man-eater tigers. Davey [7] reported that responses of captive wildlife are often species-specific and influenced by enclosure design, temperament, and prior experience. However, focused studies on rescued man-eating tigers, particularly those contrasting behaviors in the presence and absence of visitors are uncommon. This gap emphasizes the importance of studies addressing evidence-based management and welfare practices. Thus, the present study aims to provide pragmatic insights into the behavioral responses of captive man-eater tigers under varying levels of human presence.

## 2. Materials and methods

### 2.1 Study area

This study was conducted at the Central Zoo in Jawalakhel, Lalitpur, Nepal, located in the core of the Kathmandu Valley (27.67318°N, 85.31094°E). Permission to conduct this observational, non-invasive study at the Central Zoo, Jawalakhel was obtained from the National Trust for Nature Conservation (NTNC), Nepal, through the administration of the Central Zoo, Jawalakhel. No additional permits were required as the study did not involve animal handling or invasive procedures. The Central Zoo is the oldest zoological garden in Nepal. Over one million visitors come to enjoy the displays at the Central Zoo annually [8], making it the most frequently visited zoological garden in Nepal. The Rana Prime Minister, Juddha Shumsher J.B. Rana, founded the private animal collection in 1932. It was later handed over to the National Trust for Nature Conservation (NTNC) for management and development in 1995 [9]. Despite Nepal’s significant wild tiger population (estimated at 355 individuals in the 2022 national census, [10], captive tigers remain a major attraction for visitors.

There are two field enclosures that provide housing for the tigers; each is approximately 500 m² in size with trees, shady platforms, water pools, and scratching logs to encourage natural behaviors. These enclosures have chain-link fencing with small glass windows to provide better viewing for the public. The viewing area permits visitors to stand 3 feet from the fence line and 15 feet above the enclosures. There is also a smaller, off-exhibit holding area attached to the main enclosure which provided a holding cage for tigers not on exhibit. When housed in the off-exhibit enclosures, the tigers remained within potential auditory and olfactory range of one another, but visual contact was not possible. The surrounding houses exhibit rhinoceros (*Rhinoceros unicornis*), wild water buffalos (*Bubalus arnee*) and primates that contribute to constant background noise and human activity including both staff and visitors. The combination of confined space and persistent visitors presence makes the site ideal for examining how the presence of visitors affects the behavior of captive tigers [6].

### 2.2 Study population

The study population consisted of six adult tigers (four males and two females) of which four (two males and two females) were selected as the subject sample for observation (Table 1). Two tigers were excluded because one was not on public display due to health conditions and the other was unavailable during the data collection period. All four subject tigers were confirmed man-eaters and had been rescued from the wild and housed at the zoo for periods ranging from 1 to 6 years. During the study, the four subjects were typically displayed in two-week rotation period, with either a single male or a male-female pair housed in each on field enclosure, with the non-displayed tigers held in the smaller off-exhibit enclosure. The observations were based on instantaneous scans conducted every five minutes and the observer recorded the behavior of each tiger sequentially during each scan point, allowing all individuals present in the enclosure to be monitored by a single observer. The tigers were fed approximately 5 kg of buffalo meat once a day at 15:00, six days per week, with one day of fasting (Friday). Tigers were observed during routine conditions, including fasting days. However, feeding-related anticipatory behaviors were not separately quantified. Feeding was carried out by zookeepers on a regular schedule so as to minimize variation in feeding-related behaviors.

**Table 1:** List of tigers at the Central Zoo with their given name, age and sex.

S.N	Tiger’s name	Age (years)	Sex	Origin	Years in captivity
**1**	**Pratap**	11	M	Pratappur, Parsa National Park	6
**2**	**Jagati**	9	F	Bayarghara, Bharatpur	5
**3**	**Maharaj**	12	M	Chitwan National Park	5
**4**	**Maharani**	6	F	Shuklaphata National Park	1
5	Bangey	20	M	Chitwan National Park	9
6	Baghora	14	M	Bardiya National Park	4

Note: Names in bold indicate the study subjects

### 2.3 Data collection

Behavioral observations were conducted using instantaneous scan sampling [11] on the four study individuals during the summer (April, 2024) and monsoon (July, 2024) seasons. The monsoon season in Nepal typically lasts from mid-June to late September. This brings warm temperatures and heavy rain, with the highest rainfall usually occurring in July and August. April is the peak, vibrant month in Nepal featuring warm, sunny days [12]. All four tigers were observed during each observation session. At every 5-minute scan interval, the observer recorded the behavior of each individual tiger separately by sequentially scanning all enclosures. Thus, behavioral data were collected simultaneously for all four tigers throughout the 15-day sampling period in each season. Observation periods covered all peak visitation ’s hours (12:00–14:00), off hours (09:00–10:00 and 17:00–18:00) and low visitation hours (10:00–11:00 and 16:00–17:00). These time periods were selected to represent peak hours, off hours and hours with low visitors’ numbers based on the zoo schedule. All data collection was conducted by the first author using the instantaneous scan sampling method. Behaviors were recorded at 5 minutes interval using a pre-defined ethogram with partial modification adding stereotypic behavior (Table 2) developed from published literature [13] and preliminary zoo observations. Each observation session lasted 2 hours resulting in 24 scan samples per session and a total of 72 scans per individual per day. The ethogram was developed based on published zoo- behavior protocols and consulting with the experts from the zoo.

**Table 2:** Ethogram of behaviors recorded study period with their description.

S.N.	Behavior	Description
1	Resting	The tiger was awake but inactive, typically lying down or sitting quietly without movement.
2	Sleeping	The tiger was lying down with eyes fully closed, showing no signs of alertness.
3	Pacing	The tiger repeatedly (at least 3 times) walked along a fixed path, often indicating stress or agitation.
4	Feeding	The tiger actively consumed food or displayed feeding-related behaviors.
5	Vocalizing	The tiger produced audible sounds such as roaring or other calls.
6	Grooming	The tiger engaged in self-maintenance behaviors such as licking or scratching itself (self-grooming), as well as grooming directed toward other tigers (allogrooming). Grooming behaviors generally indicate comfort and a relaxed mental state.
7	Aggression	The tiger exhibited hostile or threatening behaviors toward conspecifics or humans, including growling, snarling or charging.
8	In Pool	The tiger is partially or fully immersed in water within the enclosure, often used for cooling or play.
9	Drinking	The tiger actively consumes water, typically by lapping it with its tongue.
10	Chewing	The tiger engages in chewing movements which may include processing food or manipulating objects.
11	Smelling	The tiger uses its sense of smell to investigate the environment, often by sniffing objects, the ground, or air.
12	Hiding	The tiger positions itself in sheltered or less visible parts of the enclosure, often to avoid disturbance or for comfort.
13	Scratching	The tiger uses its claws to scratch surfaces, which can serve to mark territory, stretch muscles, or remove old claw sheaths.

To complement direct observation, continuous CCTV footage was reviewed by the primary observer. Both field enclosures were covered by dual-lens high-resolution cameras (Himoobie Wifi Camera Cp6) which provided full coverage of the enclosure, enabling detection of rare or subtle behaviors that may have been missed during live observation. This method also was particularly effective for identifying stereotypic behavior such as pacing and excessive grooming. The data from the cameras were used to observe the tigers in the area of the enclosure which were not clearly visible from the vantage point where the instantaneous sampling data was taken. The footage was used to enhance data accuracy and serve as a secondary verification tool to ensure consistency, especially during high visitor periods when real time observation could be obstructed.

### Visitor presence

The level of visitor presence was distinguished based on threshold numbers of visitors present in the field enclosure view area (40m^2^) during the observation period. Threshold numbers were based on preliminary observation and space capacity for viewers in the viewing area. The level of visitor presence was assigned to three categories:

i. Low Visitor Presence: defined as 25 or fewer visitors present at the viewing platform during the sampling.
ii. High Visitor Presence: defined as more than 25 visitors present at the viewing platform during the sampling time.
iii. No Visitors: defined as no visitors present at the viewing platform during the sampling time.

### 2.4 Ethics statement

There was no direct handling or intervention with the animals during this study. The study protocol was reviewed and approved by the Central Zoo. This study employed exclusively non-invasive methodologies, and no direct handling, capture, or physical restraint or interaction with wildlife occurred during the course of the study. All research activities were conducted in strict accordance with the ethical guidelines and legal requirements for wildlife research in Nepal. Prior to data collection, official research approvals were obtained from the Central Zoo, Nepal and National Trust of Nature Conservation (Permission Letter Ref. No.: NTNC/CENTRALZOO/0218). These approvals ensured that the study complied with national conservation regulations and minimized any potential disturbance to wildlife and their habitats.

### 2.5 Data analysis

All behavioral data were categorized into several primary activity categories, including grooming, sleeping, aggression, vocalizing, pacing, smelling, scratching,in pool, hiding, drinking, chewing, and resting. Data were entered and analyzed in R Studio [14]. Descriptive statistics were computed, and chi-square tests were applied to assess relationships among behavioral categories, number of visitors, and seasonal variations. The sex variable was included as a fixed categorical factor to examine whether behavioral frequencies differed between males and females across observation periods. The graphical representations and behavioral summaries were generated using the ggplot2 package in R [15].

## 3. Results

### 3.1 Overall behavioral patterns

**Fig 1:**
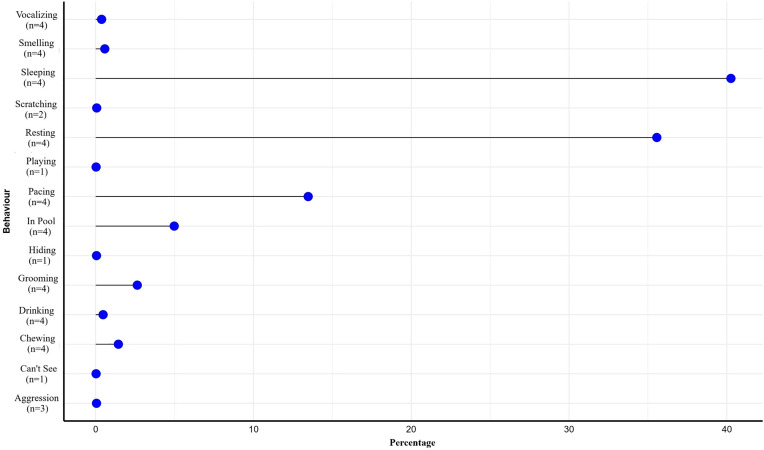
Overall activity budget of captive tigers at Central Zoo with n representing number of tigers showing respective behavior.

All four subject tigers were predominantly inactive across all seasons, with sleeping (40.3%) and resting (35.6%) constituting the majority of observed behaviors. Active behaviors such as pacing (13.5%), pool use (4.98%), and grooming (2.64%) were relatively infrequent. Other activities: chewing, drinking, aggression, hiding, scratching, playing, and vocalizing were negligible (Fig 1). Sleeping and resting combined accounted for over 75% of all recorded behaviors, reflecting their largely sedentary activity budget in captivity. Grooming behavior of the tigers showed light increase from 2.12% in the absence of visitors to 2.91% during visitor presence. The most pronounced behavioral change involved pool use, ranging from 0.86% during visitor absence to 7.13% in the presence of visitors.

### 3.2 Seasonal variation in behaviors

**Fig 2:**
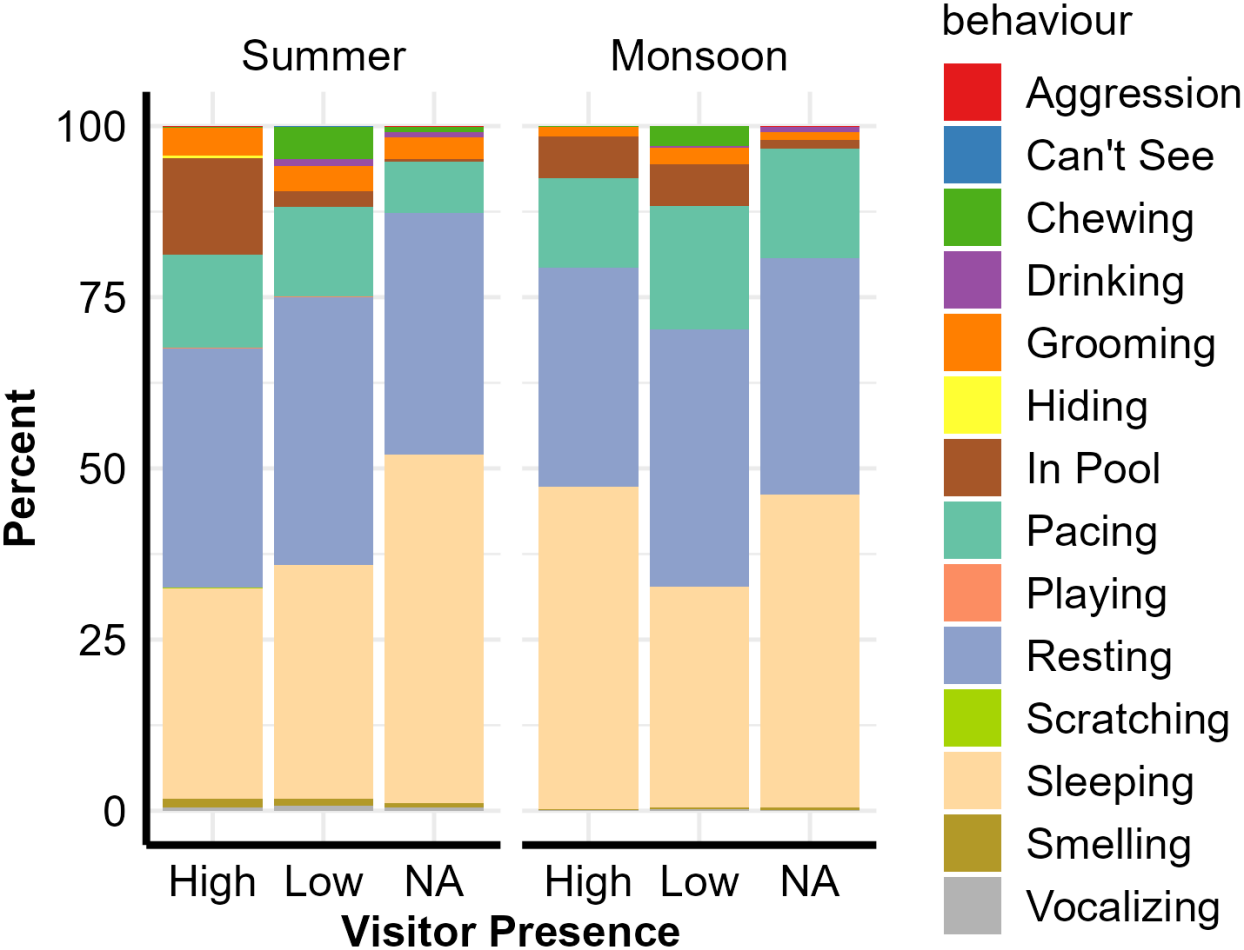
Seasonal and visitor-presence variation in tiger activity.

Seasonal behavior patterns varied significantly between visitor presence and seasons (χ² test, p < 0.001). Resting (monsoon: 34.6%; Summer: 36.4%) and sleeping (monsoon: 41.9%; mummer: 38.7%) dominated across all conditions, but activity profile differed seasonally. During summer, tigers engaged more frequently in pool use (summer: 5.5%; monsoon: 4.4%), whereas during the monsoon season, pacing (monsoon: 15.7%; summer: 11.3%) and other active behaviors were more pronounced (Fig 2). GLM analysis confirmed increased pacing during the monsoon (Visitors Low: β = 0.38, p < 0.001; Visitors No: β = 0.24, p = 0.037), while in summer, visitor density had little effect (interactions: Visitors Low: Season Summer β = −0.43, p = 0.008; Visitors No: Season Summer β = −0.91, p < 0.001).

Periods without visitors were characterized by markedly fewer stress-related behaviors, suggesting reduced external disturbance during low-visitation times. Under high visitor presence, tigers displayed greater behavioral diversity, including aggression, pacing, and hiding, whereas under low visitor presence, inactive behaviors (resting and sleeping) predominated.

### 3.3 Sexual differences in behaviors

Significant sex-related differences in behavioral expression were identified under both conditions (p < 0.05). While sleeping and resting were the dominant behaviors for both sexes, male tigers devoted a greater proportion of time to sleeping, particularly in absence of visitors. Females, by contrast, exhibited more resting and pacing behaviors with the highest pacing observed during low visitor presence (16% for female vs 15% for male).

**Fig 3:**
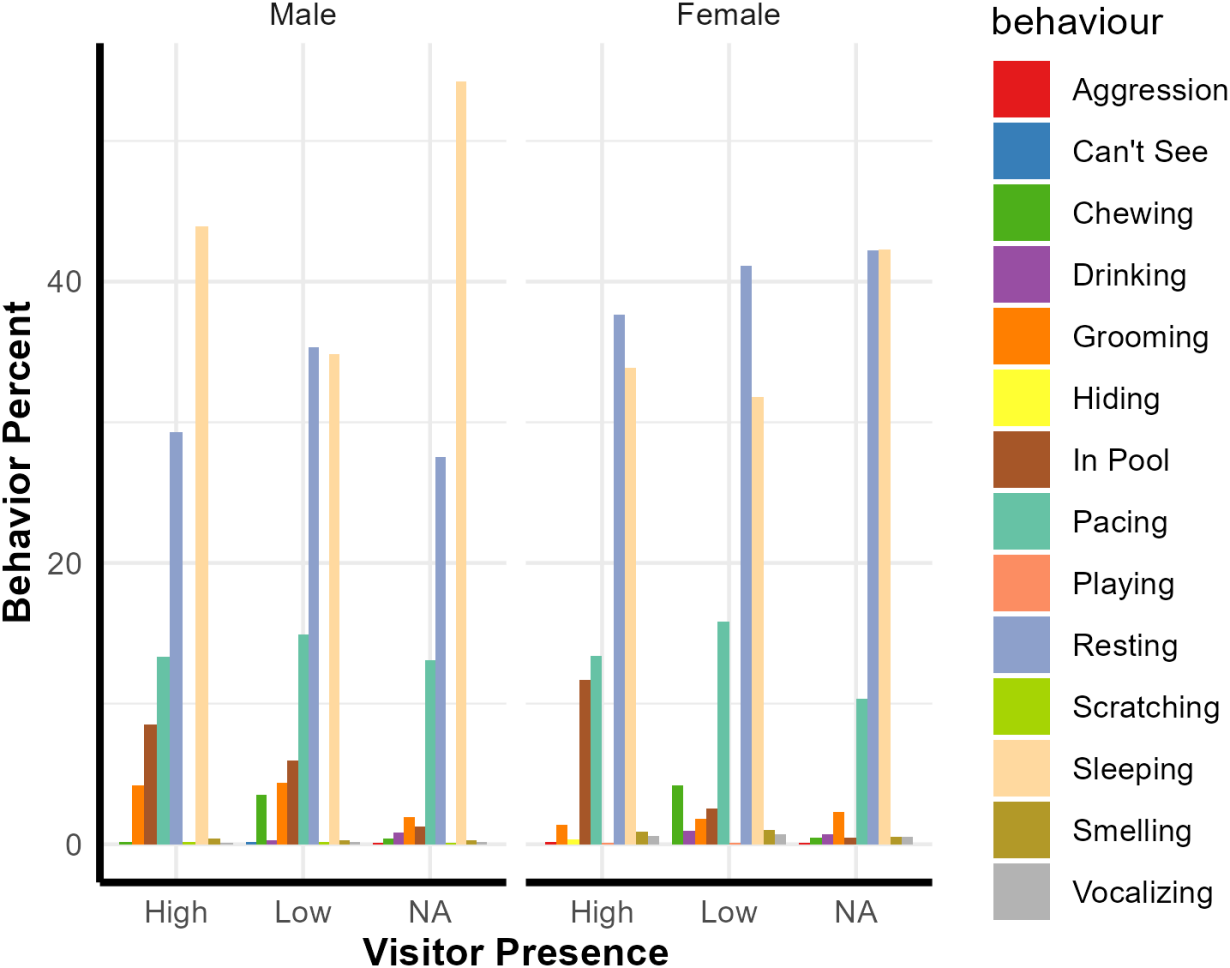
Behavioral differences between male and female tigers by level of visitor presence.

During the study period both sexes were recorded displaying increased pacing behavior during low visitor presence. Other active behaviors were generally higher during high visitor presence. Female tigers demonstrated higher rates of pacing than males across visitor conditions (Fig 3).

### 3.4 Behavioral differences by duration of captivity

**Fig 4:**
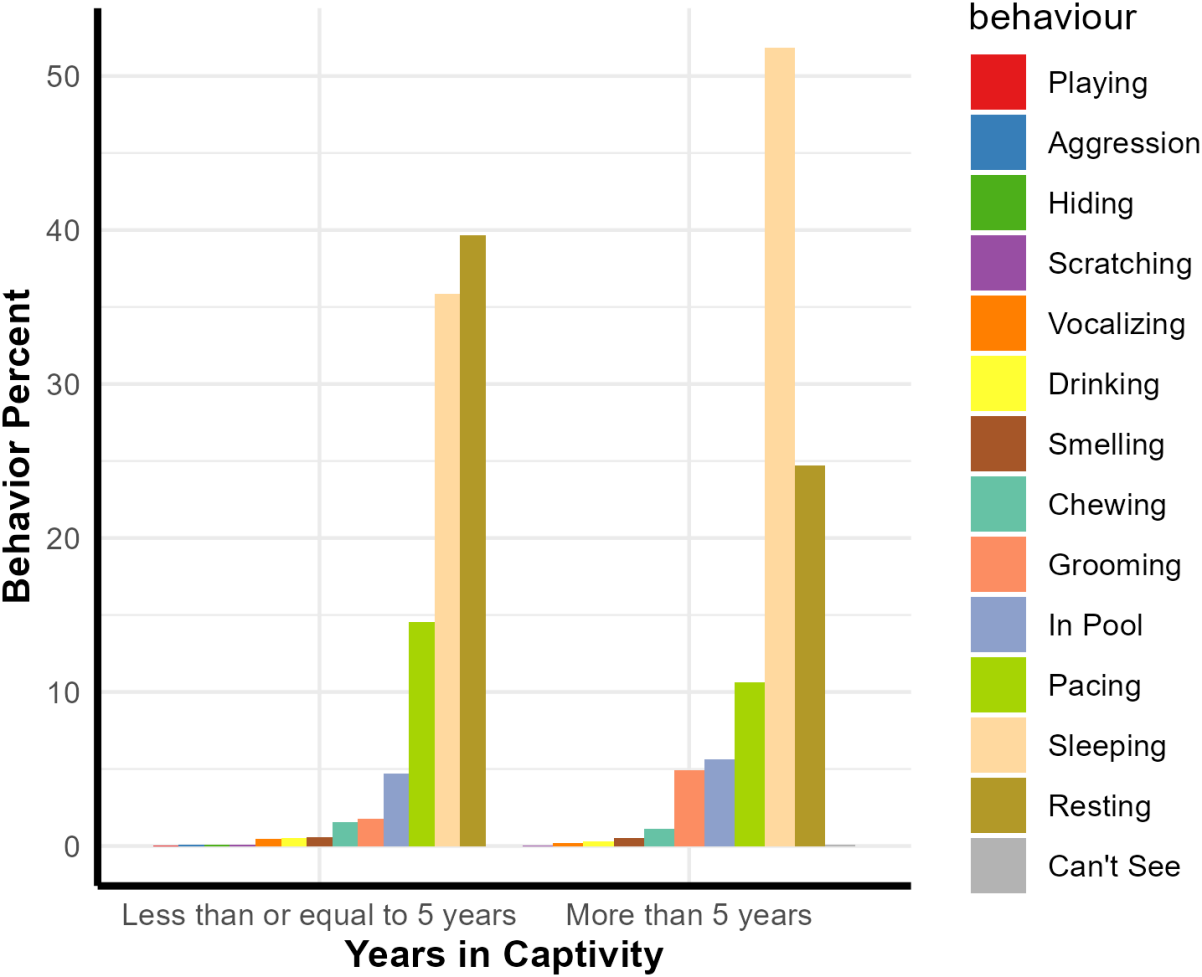
Behavioral variation by duration of captivity.

The duration of captivity significantly influenced behavioral expression (p < 0.001). Resting and sleeping remained predominant among all individuals. The tigers held in captivity for less than or equal to five years were more active and allocated a greater percentage of their time budget to active behavior including pool use (4.7%) and showed higher pacing (14.6%). In contrast, long-term captive tigers held for more than 5 years exhibited higher levels of inactivity, spending more time resting (24.7%) or sleeping (51.8%) with limited behavioral variation (Fig 4).

### 3.5 Stereotypic behaviors of captive tigers

The subject tigers displayed several stereotypic behaviors including pacing, chewing and vocalizing (Table 3). These repetitive, purposeless behaviors typically occurred in the absence of external stimuli and were more frequent during the presence of visitors. The results indicate a link between visitor presence and heightened stress among the tigers within the captive environment (Fig 3).

**Table 3:** Stereotypic behaviors observed in the tigers at the Central Zoo.

Stereotypic Behaviors	Description of the stereotypic behavior
Pacing	Repetitive locomotion along a fixed and invariant path (back-and-forth or route-tracing patterns such as figure 8) performed for at least three consecutive cycles without an apparent goal or environmental stimulus. A bout of pacing was considered to begin when such repetitive movement was initiated and to end when the individual ceased the pattern or changed to a different behavior for more than 10 seconds.
Chewing	Oral manipulation of non-food objects such as enclosure fixtures, twigs or enrichment items. This behavior may occur in both naturalistic contexts (e.g., exploration or enrichment interaction) and as a potential stereotypic or stress-related behavior depending on context. Self-directed oral behaviors (e.g., fur or body biting) were not recorded separately in this study.
Vocalizing	Any audible sound produced by the tiger, including roars, moans, growls, or chuffing. This behavior may occur in multiple contexts such as communication with conspecifics, response to environmental stimuli, anticipation of feeding, or other internal states. Vocalizations were recorded without assigning specific functional context.

Stereotypic behaviors such as pacing, vocalizing and chewing occurred more frequently during periods with visitors’ presence compared to periods with no visitors, suggesting that visitor presence and enclosure constraints may contribute to heightened stress and the expression of stereotypy (Table 4). Specifically, pacing (mean difference = 86.75, p = 0.039) and chewing (mean difference = 25.75, p = 0.005) increased significantly during high visitor periods, while vocalizing showed a slight, non-significant increase (mean difference = 3, p = 0.173).

**Table 4:** Effect of visitor presence on stereotypic behaviors of the tigers at the Central Zoo.

Behavior	Mean Difference (High – No)	p-value (t-test)
Pacing	86.75	0.039
Vocalizing	3	0.173
Chewing	25.75	0.005

## 4. Discussion

This study examined the behavior of wild tigers housed at the Central Zoo, Jawalakhel, focusing on the effects of the visitor presence, sex, and duration of captivity. The results revealed significant behavioral variations across visitor-presence levels and seasons, with strong evidence that visitor presence acts as a behavioral stressor influencing activity budgets and stereotypic behavior expression. The tigers showed elevated levels of pacing and drinking and alertness during high visitor presence, while resting and sleeping-dominated during off-peak hours. Such a pattern aligns with previous findings that visitor presence can act as a stressor for captive felids [16]. The heightened activity and vigilance may result from increased ambient noise, movement and new stimuli. Occasional aggressive behavior displays likely represent frustration or defensive responses to perceived threats. Although resting was one of the most common behaviors observed in the tigers, it should be interpreted carefully in terms of welfare. In captive felids, high levels of resting do not always indicate a good welfare state. In some cases, reduced activity can also reflect boredom, low stimulation, or other underlying welfare concerns. Therefore, both increased resting and behaviors like pacing may reflect potential welfare issues, depending on the environment and individual context.

Sexual dimorphism in behavioral expression was evident. Females were generally more exploratory and exhibited higher pacing rates, whereas males were predominantly inactive [17–19]. However a study by Zhen-sheng et al., [20] at Hangzhou Safari Park found males to be more active, indicating that differences may depend on individual temperament, enclosure design, and management practices. In this study, the females’ heightened activity during visitor peaks may reflect greater environmental reactivity or reduced tolerance to disturbance. Although the females appeared to exhibit higher levels of activity, this pattern may be confounded by age and acclimatization effects. For example the youngest individual in this study was a female who had been recently introduced to captivity. Younger tigers are generally more active and exploratory. This suggest that individuals recently introduced to an enclosure may display increased movement due to novelty or incomplete acclimatization [21]. An additional factor that may have influenced the behavioral patterns observed in the youngest female tiger may be due to temporary separation from the resident male for approximately one week during the study period. The female was periodically housed with a male; however, during the study period, the male was temporarily isolated in a holding enclosure for approximately one week due to a leg injury. During this period, the female exhibited comparatively higher levels of pacing and occasional vocalization than at other times. These behaviors may reflect a response to social separation and the inability to access a familiar conspecific. The temporary separation may have influenced the female’s activity levels and social responses, thereby contributing to observed behavioral differences. Duration of captivity also emerged as a key determinant of behavior. The tiger held in captivity for shorter periods were more playful and exploratory, while long-term captives exhibited reduced behavioral diversity and increased inactivity which is consistent with the development of habituation or coping mechanisms over time [22–24]. This supports the hypothesis that prolonged confinement fosters predictable behavioral routines and reduces responsiveness to external stimuli. It is also possible that this individual exhibited heightened responsiveness to human presence due to incomplete habituation to captivity. It is important to note, however, that this comparison is based on a single individual held captive for only one year versus three individuals maintained in captive for five years or more.

The spatial distribution of visitors within different viewing sections of the enclosure was not quantified as part of this study and thus limit interpretation of localized disturbance effects. Previous studies have shown that increased number of visitors and proximity to viewing areas can influence the behavior of captive animals [25,26]. Comparable findings have been documented in other urban zoos where increased crowd density correlates with elevated vigilance and stereotypic activity [27].

The occurrence of stereotypic behaviors, such as pacing, chewing, and vocalizing, indicate potential welfare concerns associated with spatial restriction and environmental monotony [22,28]. Pacing is the most prevalent stereotypy that may represent a combination of anticipatory activity (e.g., prior to feeding) and stereotypic movement rather than solely reflecting frustrated motivation for territorial exploration which is a fundamental behavioral needed for wide-ranging carnivores. Similarly, non-nutritive chewing likely represents redirected foraging or stress-related displacement but it may also occurs a part of natural exploratory or predatory behaviors [23], while repetitive vocalizations suggest arousal or attempts at social engagement [29]. The strong association between visitor presence and stereotypic frequency underscores the influence of anthropogenic disturbance on welfare outcomes. Comparable findings in other zoological settings have demonstrated that crowd size, noise, and human proximity exacerbate stress and stereotypy in big cats [7,25,30].

These findings highlight the need for evidence-based interventions to improve the welfare of captive tigers. Management strategies should focus on environmental enrichment. This can include variable feeding schedules, movable enrichment items, and sensory stimulation to promote natural behavioral expression. The inclusion of visual barriers and retreat spaces can help mitigate the effects of visitor presence, while spatial redesign of enclosures—incorporating increased complexity and opportunities for choice—can reduce stress and promote behavioral diversity. Visitor management strategies, such as crowd control and educational outreach, can minimize direct disturbance. Implementing such welfare-oriented measures will not only improve the psychological health of captive tigers but also enhance the educational and conservation value of zoological institutions by encouraging the display of species-typical behaviors under enriched and ethically managed conditions [31,32].

Although the study provides valuable insights into the behavioral responses of captive tigers to the presence of visitors and other environmental conditions, it is limited by the sample size. As such, the findings may not be fully generalizable to larger or more diverse captive tiger populations across other institutions. Further study with larger sample sizes, multiple zoo environments and management practices would strengthen the robustness and applicability of the data.

## 5. Study limitations

The study involved only four tigers, limiting the generalizability of the results to other captive tiger populations. The individuals differed in age, sex and duration of captivity, may influenced behavioral responses independently of visitor presence. Additionally, behavioral observations were conducted within the constraints of normal zoo management practices and other uncontrolled factors such as weather conditions, enrichment schedules, husbandry activities and individual personality differences may also have affected behavior. Therefore, the observed associations should be interpreted cautiously and future studies incorporating larger sample sizes across multiple zoological institutions are recommended.

## 6. Conclusion

This study indicates that the behavior of wild tigers at the Central Zoo, Jawalakhel, varies with visitor presence, sex, and duration of captivity. The tigers exhibited increased activity, particularly pacing, during period of high visitor presence, whereas resting and sleeping were more common during off-hours. Recently captured female showed greater exploratory and pacing behavior, while males were more prone to rest and sleep. The length of captivity also played a critical role, The recently captured tiger displayed more active and variable behaviors, whereas the longer-term captives exhibited reduced responsiveness and more stereotypic patterns. These findings highlight the complex interaction between environmental stimuli, individual traits, and captivity history in shaping behavioral welfare. Variation from other studies may reflect differences in enclosure design, enrichment quality, and management practices. Collectively, the results emphasize the need for evidence-based welfare interventions particularly enhanced enrichment, visitor regulation, and individualized care to mitigate stress and promote naturalistic behavior. Implementing such measures will enhance both animal welfare and the educational and conservation outcomes of zoological institutions.
